# On the Symposium “Concept of Integrated Approaches to Testing and Assessment (IATA) Using New Approach Methods (NAMs) —Learning from the Study of Thyroid Hormone System and Developmental Neurotoxicity—”

**DOI:** 10.14252/foodsafetyfscj.D-25-00024

**Published:** 2025-09-26

**Authors:** Shigeki Yamamoto, Satoshi Asano, Tomotaka Sobue, Masahiro Tohkin

**Affiliations:** Food Safety Commission, Cabinet Office, Government of Japan, Akasaka Park Bldg 22F, 5-2-20 Akasaka, Minato-ku, Tokyo 107-6122, Japan.

**Keywords:** Integrated approaches to testing and assessment, new approach methods (NAMs), adverse outcome framework

## Abstract

Food Safety Commission of Japan (FSCJ) hosted a symposium entitled “Concept of Integrated Approaches to Testing and Assessment (IATA) Using New Approach Methods (NAMs) —Learning from the Study of Thyroid Hormone System and Developmental Neurotoxicity—” to deepen our understanding on the application of NAMs through practical examples. The symposium took place at FSCJ meeting room (Akasaka, Minato-ku, Tokyo) on December 5th, 2024, attracting a total of 21 on-site attendees as well as 153 online participants. FSCJ Chairperson Dr. YAMAMOTO gave an opening remark. In the presentation 1, Dr. AKAHORI Yumi from the Chemicals Evaluation and Research Institute, Japan (CERI), who also serves as a member of FSCJ’s Working Group on Assessment Methodology Development, presented a lecture titled “International Activities Related to NAMs.” In the presentation 2, Dr. Mary Gilbert from the US EPA gave a lecture titled “Thyroid Disruption and Neurodevelopment in an Adverse Outcome Framework Translating NAMs— Filling in Gaps.” In the Q&A session, participants discussed about the concept and definition of NAMs. In concluding the seminar, Dr. ASANO remarked that, given the active efforts to leverage NAMs abroad led by Europe and the US, FSCJ will continue to discuss various NAMs which would be accumulated internationally and their potential applications.

## 1. Introduction

Recently, global discussions have been taking place on the introduction of New Approach Methods (NAMs) for evaluating the risks of chemical substances. These discussions focus on animal welfare and the limitations of the extrapolation of results from conventional animal testing to human toxicity due to species differences.

Building on this theme, at our 2023 Symposium commemorating the 20th Anniversary of Food Safety Commission of Japan (FSCJ), it was reaffirmed that further development of NAMs under the Integrated Approaches to Testing and Assessment (IATA) framework is important to fill data gaps and enhance the precision of evaluations using existing animal data. It was also acknowledged then that such development requires collaboration with organizations abroad, such as those in Europe and the United States, highlighting the necessity of global cooperation.

FSCJ hosted a symposium entitled “Concept of Integrated Approaches to Testing and Assessment (IATA) Using New Approach Methods (NAMs) —Learning from the Study of Thyroid Hormone System and Developmental Neurotoxicity—” to deepen our understanding on the application of NAMs through practical examples. The symposium took place at FSCJ meeting room (Akasaka, Minato-ku, Tokyo) on December 5th, 2024, attracting a total of 21 on-site attendees as well as 153 online participants. The outline of each segment is as follows.

## 2. Opening Remark

Summary of FSCJ Chairperson Dr. YAMAMOTO’s opening remark

• Recently, there has been active discussion on the use of NAMs, a topic that FSCJ also addressed as one of the themes at our 20th Anniversary Symposium held last September.

• For this symposium, we have invited Dr. AKAHORI Yumi, a member of FSCJ’s Working Group on Assessment Methodology Development, to firstly give a lecture on the global trends in NAMs. Following this, we are also honored to have Dr. Mary Gilbert, a senior investigator from the United States Environment Protection Agency (US EPA), which is particularly focused on the development of NAMs, to present her research and findings. Through these lectures, we hope to deepen our understanding on the significance and necessity of NAMs.

• In the latter half, we will have a Q&A session moderated by Dr. AOYAMA Hiroaki, who served as a member of Expert Committee of FSCJ on Veterinary Medicinal Products until last year.

• We hope that this symposium will be helpful in considering the future applicability of NAMs.

## 3. Presentations

### 3.1 Presentation 1: “International Activities Related to NAMs” 

We were honored to have Dr. AKAHORI Yumi from the Chemicals Evaluation and Research Institute, Japan (CERI), who also serves as a member of FSCJ’s Working Group on Assessment Methodology Development, present an insightful lecture.

Dr. AKAHORI firstly explained that NAMs generally include technologies such as *in silico*, *in chemico* and *in vitro* assay methodologies. Although there is no internationally agreed definition as of yet, NAMs also encompass evaluation methods such as IATA which integrates these related methodologies.

Secondly, with respect to international trends, Dr. AKAHORI noted that many organizations in Europe and the US, both governmental and non-governmental, are currently accelerating the development of NAMs. She also mentioned that OECD and other organizations are systematically organizing findings regarding risk assessment methods based on accumulated experiences and identifying issues related to risk assessment using NAMs.

Lastly, although there are differences between Japan and Europe or the US in legal background and human resources for safety assessments, it was also noted that NAMs tend to be incorporated into international rules, such as OECD Test Guidelines, and that this trend is likely to continue.

### 3.2 Presentation 2: “Thyroid Disruption and Neurodevelopment in an Adverse Outcome Framework Translating NAMs— Filling in Gaps”

We were honored to have a presentation by Dr. Mary Gilbert of the US EPA. As an example among assessments related to developmental neurotoxicity (DNT), Dr. Gilbert shared findings from research on the effects of endocrine-disrupting chemicals by applying NAMs related to the assessment of the thyroid system.

The progress of this research is based on a growing demand from regulatory authorities for risk assessment methods that are more efficient and reflect the latest scientific knowledge, because a vast number of risk assessments for chemicals is required.

In the current OECD Test Guidelines, the Adverse Outcome Pathway (AOP) from the decrease of thyroid hormone (TH) levels in serum to DNT is established. However, it is also recognized that there are gaps in understanding the intermediate mechanisms involved in this pathway.

Dr. Gilbert explained through her presentation that animal models were established to assess the effects of decreased serum TH levels on fetuses. These models enabled the identification of structural defects in the brain and downregulation of gene expression. She further elucidated that these defects serve as novel markers correlated with functional changes in physiology and behavior.

Her presentation suggested the possibility that elucidating the mechanisms of DNT and identifying related markers could boost efficient assessments that maintain the necessary reliability based on sufficient data.

## 4. Q&A Session and Discussions

Participants discussed about the future of NAMs and their practical applications in risk assessments. Our distinguished speakers shared examples on NAMs data complement guideline testing results in the US and other countries.

## 5. Closing Remark

In concluding the seminar, Dr. ASANO remarked that, given the active efforts to leverage NAMs abroad led by Europe and the US, FSCJ will continue to discuss various NAMs which would be accumulated internationally and their potential applications.

